# Perception of soft mechanical stress in Arabidopsis leaves activates disease resistance

**DOI:** 10.1186/1471-2229-13-133

**Published:** 2013-09-13

**Authors:** Lehcen Benikhlef, Floriane L’Haridon, Eliane Abou-Mansour, Mario Serrano, Matteo Binda, Alex Costa, Silke Lehmann, Jean-Pierre Métraux

**Affiliations:** 1 Department of Biology, University of Fribourg, 10 chemin du Musée, CH-1700, Fribourg, Switzerland; 2 Department of Biosciences, University of Milan, via G. Celoria 26, 20133, Milan, Italy

## Abstract

**Background:**

In a previous study we have shown that wounding of *Arabidopsis thaliana* leaves induces a strong and transient immunity to *Botrytis cinerea*, the causal agent of grey mould. Reactive oxygen species (ROS) are formed within minutes after wounding and are required for wound–induced resistance to *B. cinerea*.

**Results:**

In this study, we have further explored ROS and resistance to *B. cinerea* in leaves of *A. thaliana* exposed to a soft form of mechanical stimulation without overt tissue damage. After gentle mechanical sweeping of leaf surfaces, a strong resistance to *B. cinerea* was observed. This was preceded by a rapid change in calcium concentration and a release of ROS, accompanied by changes in cuticle permeability, induction of the expression of genes typically associated with mechanical stress and release of biologically active diffusates from the surface. This reaction to soft mechanical stress (SMS) was fully independent of jasmonate (JA signaling). In addition, leaves exposed soft mechanical stress released a biologically active product capable of inducing resistance to *B. cinerea* in wild type control leaves.

**Conclusion:**

Arabidopsis can detect and convert gentle forms of mechanical stimulation into a strong activation of defense against the virulent fungus *B. cinerea*.

## Background

Plants are exposed to various forms of mechanical stress caused by rain, snow, wind, animals, pathogens or plants themselves. Such mechanical stimuli induce responses in the plant that were shown in many cases to have an adaptive value [1]. A classical example is the response of trees to wind that results in shorter and thicker trunks. Reaction or compression wood is an anatomical consequence of sensing mechanical stress with subsequent lignification of cell walls [2,3]. Plants also respond to a more delicate mechanical stress referred to as touch that leads to nastic or tropic responses (thigmonasty or thigmotropism). Classical examples include the folding of *Mimosa pudica*’s leaflets, the leaf closure of the Venus fly trap or the coiling of tendrils [4]. Such stimuli lead to visible responses such as a reorientation of organs or changes in shape allowing catching an insect or improved anchorage. The response of plants to mechanical stimuli can also be more discrete without any apparent overt changes. For example, mechanical stress associated with damage or wounds can lead to increased resistance to insects [5,6] or fungal pathogens [7-9].

A closer look at the response to wounding has shown that it induces biochemical and molecular changes often associated with subsequently induced resistance mechanisms. For instance, wounded plants produce reactive oxygen species (ROS) [10,11], undergo changes in lignification [12], JA, other hormones or wound signals [13] and exhibit changes in gene expression [5,14] that are associated with induced defense reactions.

In a previous study we have shown that wounding of *Arabidopsis thaliana* leaves induces a strong and transient immunity to *Botrytis cinerea* the causal agent of grey mould [7]. The expression of genes for camalexin biosynthesis and of glutathione-S-transferase, the activity of a MAP kinase activity and the accumulation of camalexin are primed by wounding [7]. Wound-induced immunity is independent of the major plant defense pathways involving salicylic acid, JA or ethylene, but depends on glutathione [7]. Recently, we have shown that wounding leads to the formation of ROS within minutes and ROS are required for wound–induced resistance to *B. cinerea*[10]. A strong constitutive resistance to *B. cinerea* also takes place in mutants such as *bdg* and *lacs2.3* defective in the production of a functional cuticle and displaying a phenotype of enhanced cuticular permeability [15]. Moreover, leaf surfaces treated with cutinase produced ROS and became more protected to *B. cinerea*[10]*.* Thus, increased permeability of the cuticle is linked to ROS formation and resistance to *B. cinerea*. In this study, we have further explored the responses of *A. thaliana* such as ROS and resistance to *B. cinerea* in leaves that are subjected to more gentle form of mechanical stimulation.

## Results

### SMS treatment of *A. thaliana* leaves induces ROS and resistance to *B. cinerea*

**Figure 1:**
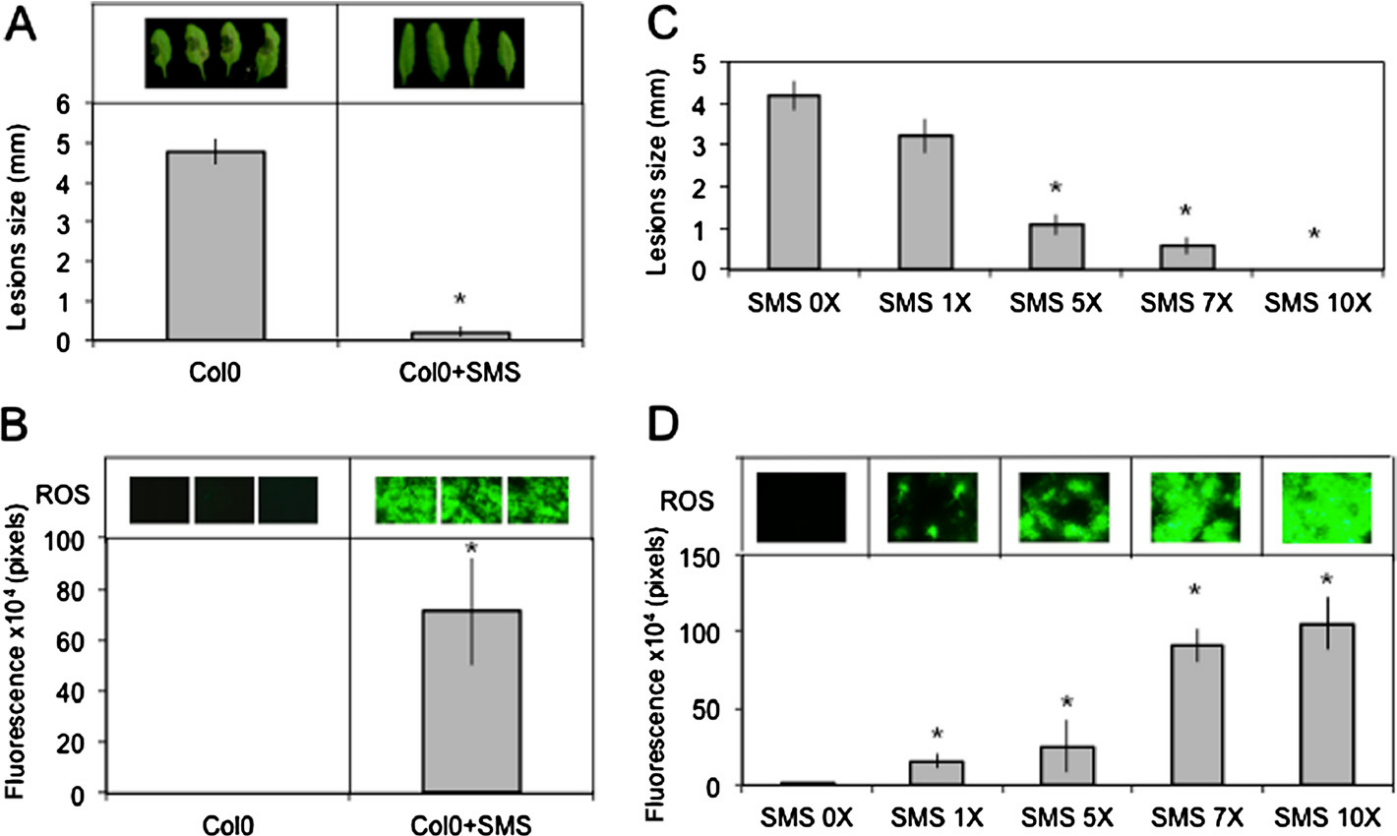
**Resistance to *B. cinerea *and ROS production in response to SMS in leaves of *A. thaliana *Col0*. *(A)** Effect of 10 SMS events on resistance of *A. thaliana* Col0 to *B. cinerea* (n = 48; ±SE). Four representative pictures of necrosis caused by *B. cinerea* were placed above each histogram as a visual illustration. **(B)** Quantification of ROS production in leaves of *A. thaliana* Col0 after 10 events of SMS (n = 12; ±SD). ROS were determined immediately after SMS. Three representative images of the fluorescent leaf surface were placed above each histogram as a visual illustration. **(C)** Dose–response of SMS-induced resistance to *B. cinerea* and ROS accumulation in leaves of *A. thaliana* Col0. Single or multiple SMS events were carried out on leaves prior to inoculation with *B. cinerea* (n = 48; ±SE). **(D)** Dose–response of SMS-induced ROS accumulation in leaves of *A. thaliana* Col0. Single or multiple SMS events were carried out on leaves prior to quantification of ROS production (n = 12; ±SD). One representative image of the fluorescent leaf surface was placed above each histogram as a visual illustration. For all experiments in this figure, plants were kept under humid conditions after treatment and each experiment was repeated 4 times with similar results. Asterisks indicate statistically significant differences between treated samples and non-treated samples, *T*-Test (p < 0,01).

**Figure 2:**
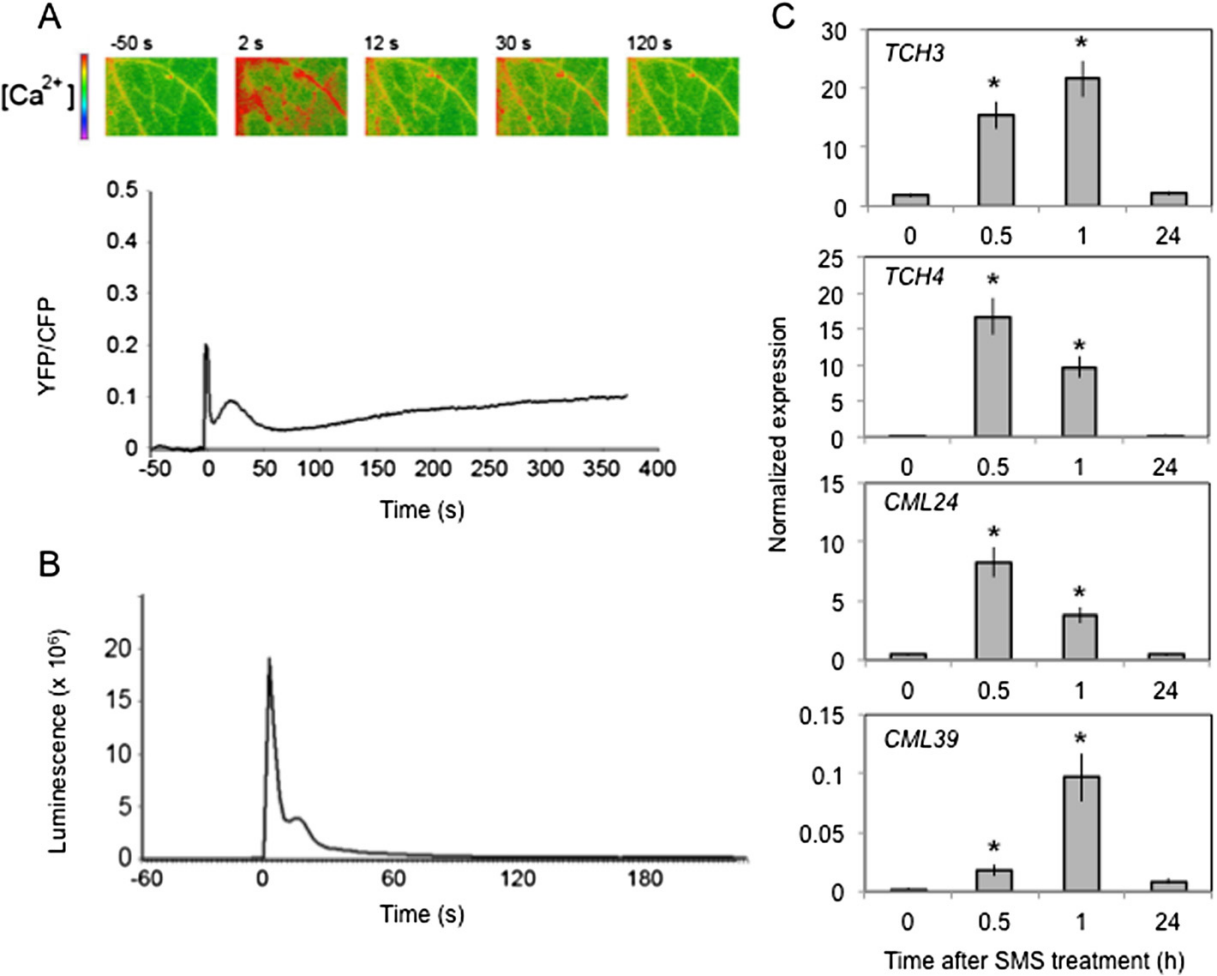
**Induction of a calcium peak and touch-induced genes after SMS. (A)** Changes in cytosolic calcium levels after one SMS event at time 0 s were monitored by FRET using the cameleon yellow protein YC3.6. The experiment was repeated 5 times, one representative time-course is presented. A visual illustration of the time course was placed above the curve at the indicated time point. **(B)** Changes in cytosolic calcium levels after five SMS events at time 0 s were monitored using the calcium sensing protein aequorin. The experiment was repeated 5 times, one representative time-course is presented. **(C)** The expression of selected touch genes was determined at various times after SMS (10×)(n = 3; ±SD). The experiment was performed three times with similar results. Asterisks indicate statistically significant differences between SMS-treated samples at different time point (0.5,1, 24 h) in comparison to non-treated samples (0), *T*-Test (p < 0,05).

**Figure 3:**
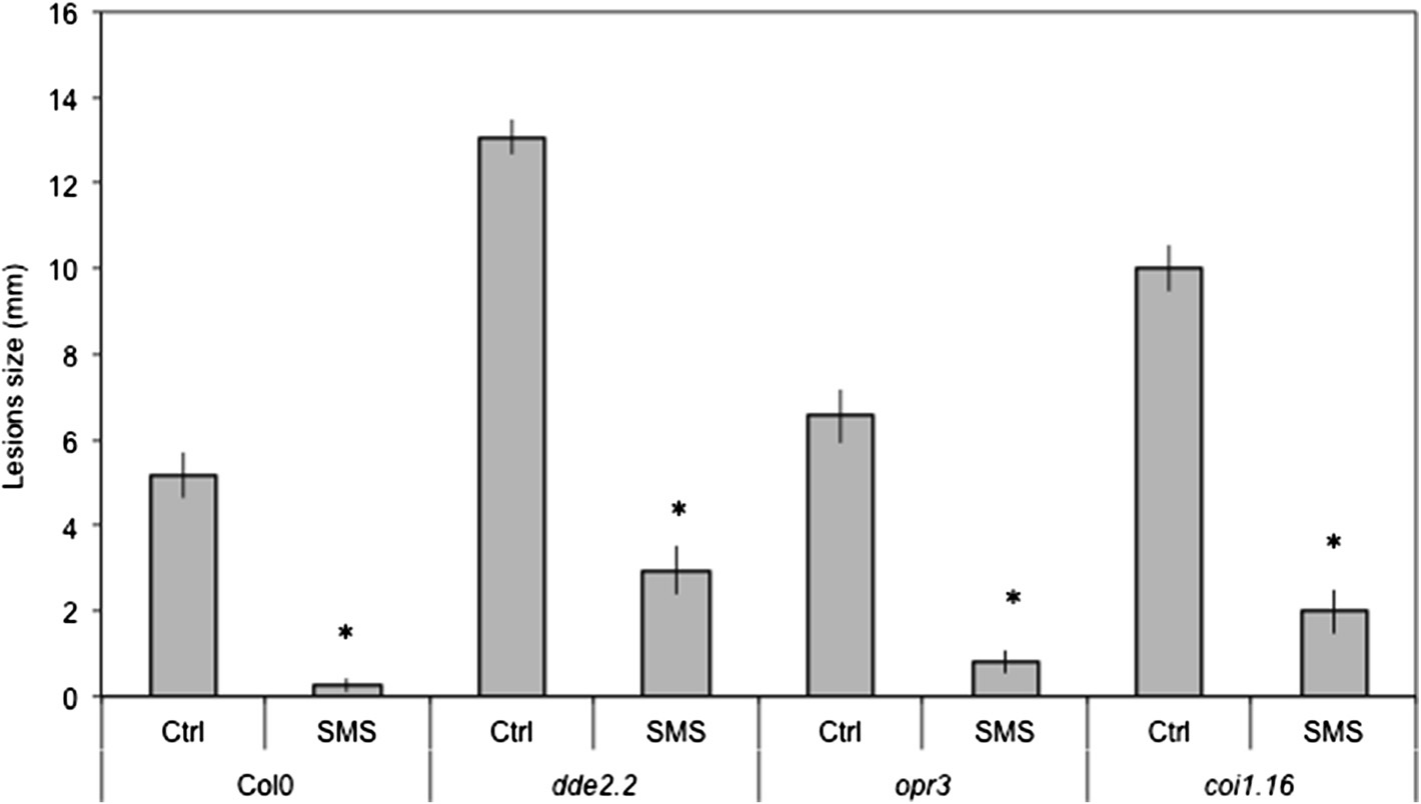
**Independence of SMS-induced resistance on jasmonic acid.** Leaves of JA mutants were SMS-treated 10 times prior to inoculation with *B. cinerea*. SMS-induced resistance to *B. cinerea* was still detected in *dde2.2, opr3* and *coi1.16* (n = 64; ±SE), the experiment was repeated twice with similar results*.* After SMS, all plants were kept under humid conditions. Asterisks indicate statistically significant differences between non-treated and SMS-treated plants for Col0 and each mutant, *T*-Test (p < 0,01).

We have treated leaves of *A. thaliana* by gently rubbing them between thumb and forefinger, a mechanical stress that is herewith referred to as a soft mechanical stimulation (SMS). The inoculation of leaves immediately after SMS with spores of *B. cinerea* led to a strong decrease in lesion size (Figure 1A). A rapid burst in ROS evidenced by a green fluorescence was observed in leaves infiltrated with 5-(and-6)-carboxy-2,7-dichlorodihydrofluorescein diacetate (DCF-DA) immediately after SMS (Figure 1B). DCF-DA detects a broad range of oxidizing reagents including H_2_O_2_ and O_2_^-^ and its use has been previously described [10]. SMS-induced resistance as well as wound-induced resistance were still detected in mutants of NADPH oxidase D (*atrboh D*) and F (*atrboh F*) as well as in the double mutant (*atrboh D/F*) meaning that others RBOH proteins are implicated in the formation of ROS (Additional file 1). The response to SMS showed a dose-dependence (Figure 1C-D). Applying only one event of SMS already lead to a small but detectable decrease in lesion size (Figure 1C) as well as production of discrete patches of green DCF-DA-fluorescence (Figure 1D). We used 10 stimulations throughout this study as this lead to the strongest effects. Unstimulated upper leaves of plants stimulated on one lower leaf were not protected against *B. cinerea* (data not shown) showing the absence of a systemic effect. Moreover, wearing latex gloves was equally effective for SMS-induced resistance (data not shown). The SMS-induced resistance to *B. cinerea* was transient: when plants were inoculated 8 h after SMS, about 50% of the resistance was lost and 24 h after SMS, plants were fully susceptible (Additional file 2). SMS was followed by a rapid change in intracellular calcium as detected using both yellow cameleon- or aequorin-expressing plants [16] (Figure 2A, B). The expression of so-called touch genes previously associated with mechanical stimulation such as *TCH3*, *TCH4*, *CML24* and *CML39*[17] was also induced 0.5 to 1 h after SMS treatment (Figure 2C). The effect of SMS could still be observed in mutants of JA biosynthesis and signaling (Figure 3). The ethylene mutant *ein2-1* also responds to SMS (Benikhlef, 2010; PhD thesis, University of Fribourg).

### SMS is not accompanied by cellular damage

**Figure 4:**
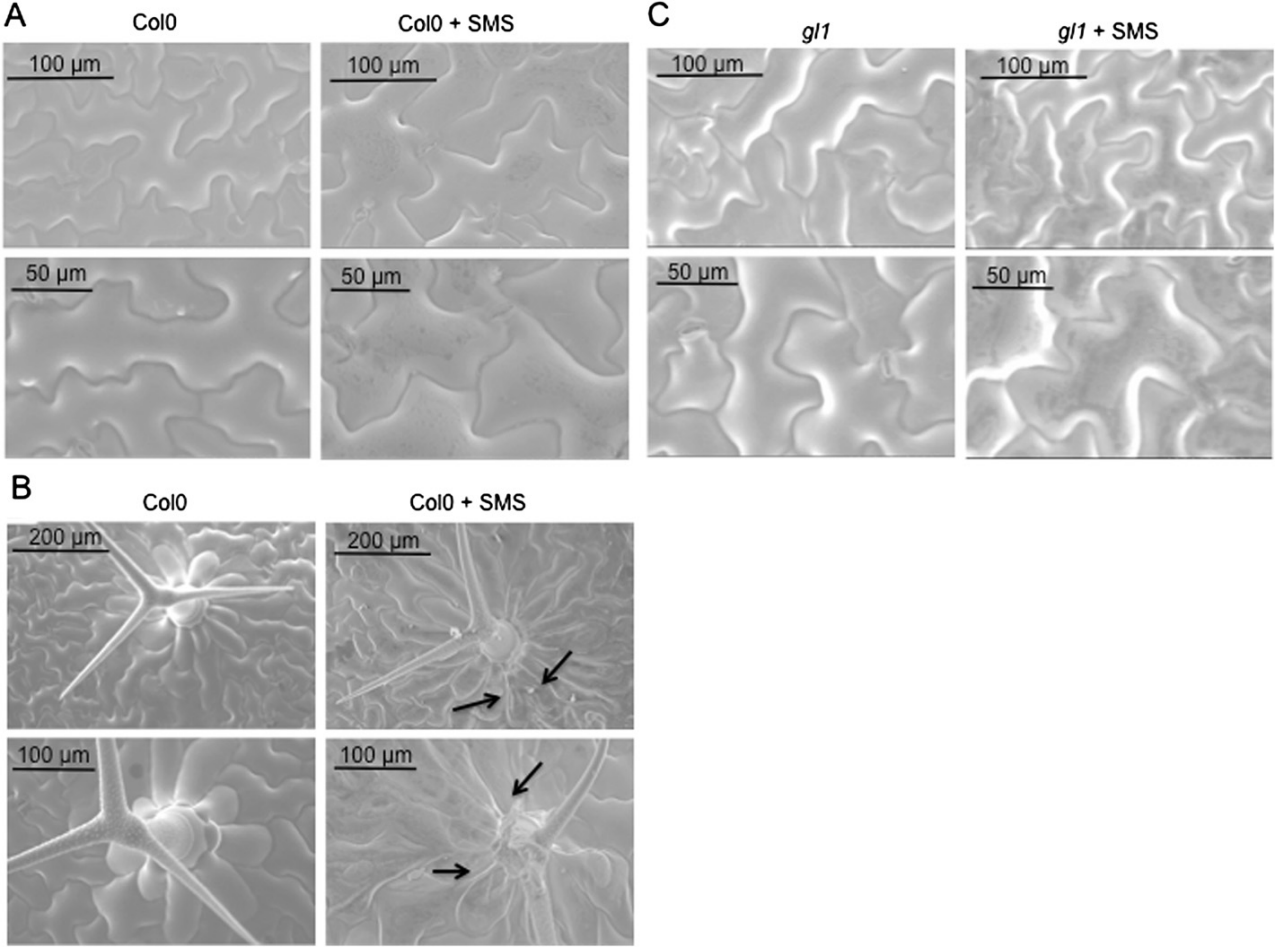
**Integrity of epidermal cells after SMS treatment visualised by SEM. (A)** Surfaces of leaves *of A. thaliana* Col0 were observed by SEM after SMS (10×). **(B)** Damage to trichomes after SMS (10×) compared to non-treated leaves. **(C)** Surfaces of leaves of the glabrous mutant *gl1* of *A. thaliana* Col0 observed by SEM after SMS (10×). All observations of this figure were repeated 25 times on 5 different leaves. Representative samples are shown.

**Figure 5:**
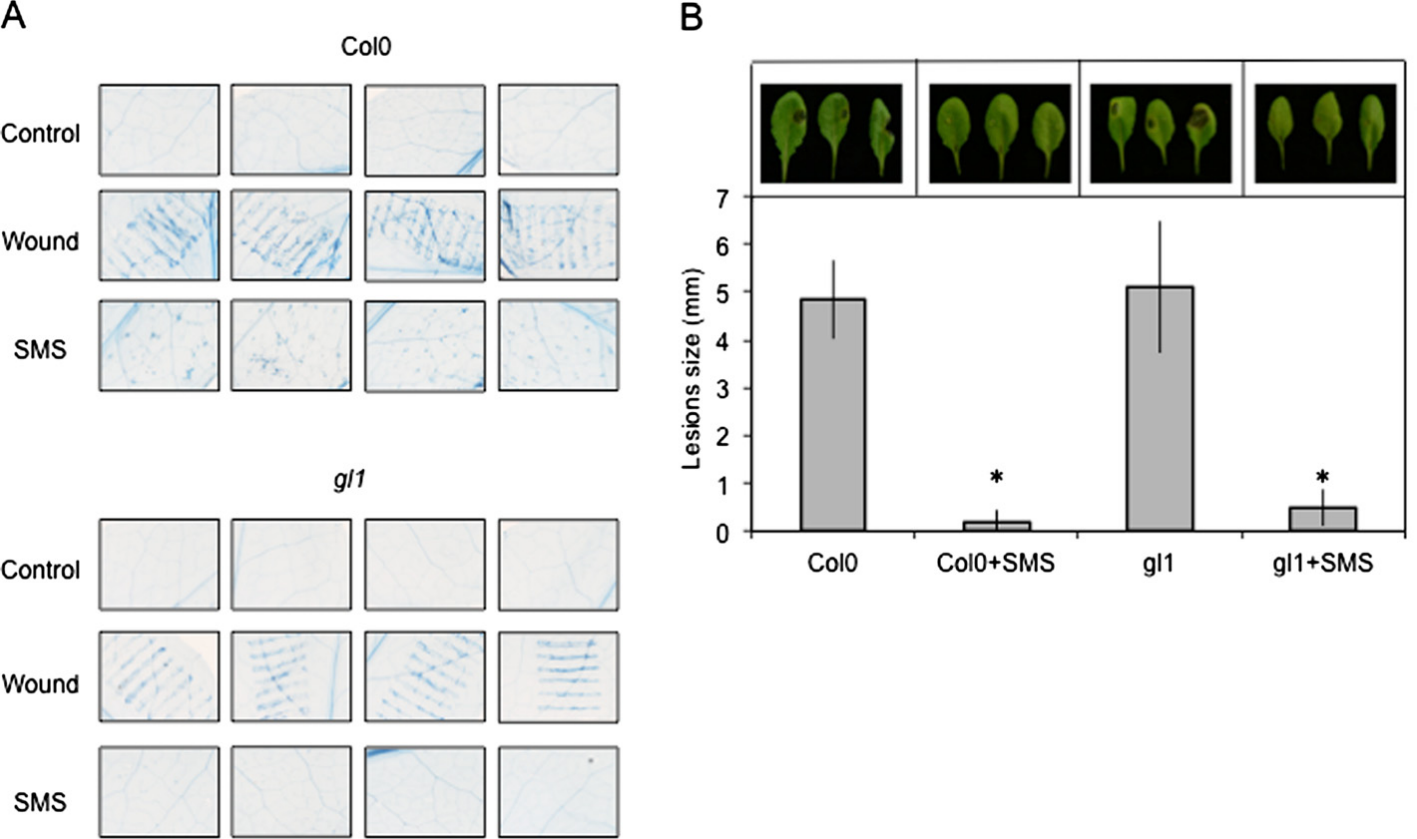
**Integrity of epidermal cells after SMS treatment visualised by vital staining. (A)** Leaves of *A. thaliana* Col0 or the glabrous *gl1* mutant were treated by SMS (10X) or wounded with forceps and visualized after 1 day by using Trypan blue staining. All plants were kept under humid conditions after treatment prior to trypan blue staining. Observations were made 2 times on 6 different leaves. Representative samples are shown. **(B)** Resistance to *B. cinerea* in response to SMS in *gl1* mutant compared to Col0 plants (n = 96; ±SE). After *B. cinerea* inoculation, all plants were kept under humid conditions. The experiment was repeated 2 times. Asterisks indicate statistically significant differences between non-treated leaves and SMS-treated leaves, *T*-Test (p < 0,01).

We next examined the occurrence of overt wounding after SMS, since wounding was shown previously to strongly affect resistance to *B. cinerea*[7]. Macroscopic signs of wounding were not visible on SMS-treated leaves. Nevertheless we assessed the effect of SMS on the cell surface by scanning electron microscopy (SEM) of live leaf surfaces as well as vital staining using trypan blue. SMS-treated epidermal cells looked perfectly turgid when viewed under the SEM (Figure 4). The waxy surface of some cells appeared slightly affected but the cells themselves retained turgidity. Some trichomes and cells at their base displayed damage (Figure 4B). When SMS-treated leaves were stained using trypan blue, a vital dye that marks the presence of dead cells, epidermal cells were essentially intact 1 h after treatment with the exception of isolated cell groups that stained in blue at the basis of trichomes (Figure 5A), in agreement with observations made using the SEM directly after SMS. Thus, SMS did not lead to massive cellular damage compared to wounding with forceps that was observed previously [10]. The question remains if the damaged cells at the basis of the trichomes might constitute a sufficiently strong wound stimulus to induce ROS and resistance. This question was approached using the trichome-less glabrous *gl1* mutant of Arabidopsis [18]. SEM images of leaf surfaces of the *gl1* mutant were compared to WT plants and in both plants cells retained turgidity after SMS (Figure 4). No damaged cells were observed using the vital stain trypan blue in *gl1* mutant after SMS (Figure 5A). In fact, the untreated *gl1* mutant is as susceptible to *B. cinerea* as WT plants and after SMS, *gl1* displayed resistance to *B. cinerea* to the same extent as the wild type (Figure 5B). Thus, SMS induced resistance independently of the presence of trichomes and SMS-induced resistance to *B. cinerea* is not based on wounding of cells.

### Alterations in cuticular permeability and SMS

**Figure 6:**
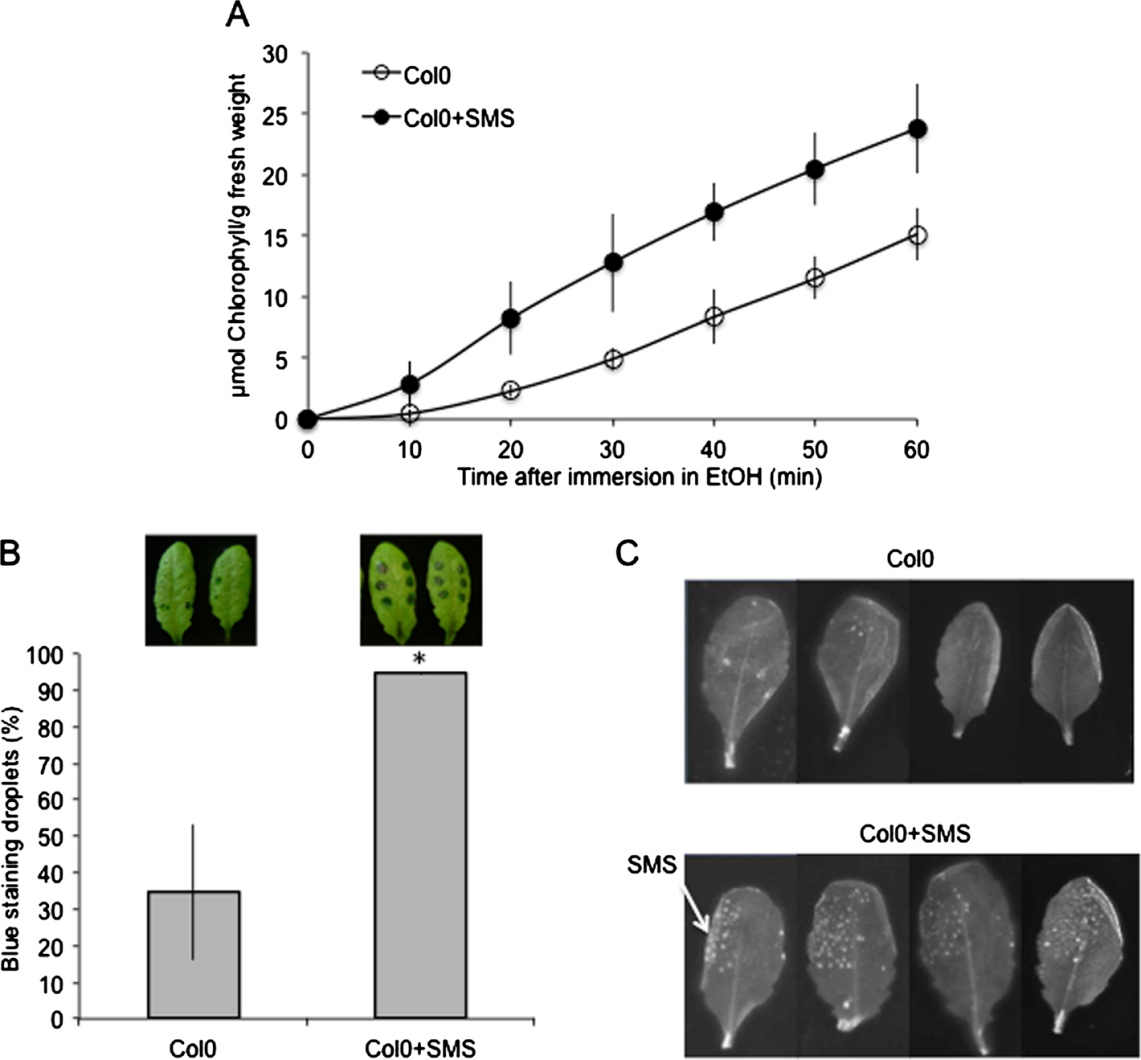
**The effect of SMS on the permeability of *A. thaliana *Col0 leaves. (A)** Permeability of the cuticle measured by chlorophyll leaching in SMS-treated leaves compared to controls (n = 3; ±SD); the experiment was carried out 2 times, one typical result is represented. **(B)** Droplets of toluidine blue were placed on the both sides of leaf surface for 2 h in high humidity then the leaf surface was rinsed with water. The blue stain that remains attached to the cell wall is indicative of a permeable cuticle. The percentage of droplets stained in blue relative to the total inoculated droplets was calculated (n = 3; 90 droplets per experiment; ±SD). Two representative pictures of leaves stained with toluidine blue were placed above each histogram as a visual illustration. Asterisks indicate statistically significant differences between SMS-treated and non-treated Col0 leaves, *T*-Test (p < 0,01). **(C)** leaves were bleached overnight in ethanol then stained with Calcofluor white that binds to cellulose, and viewed under UV light. Calcofluor staining to the leaf is indicative of a permeable cuticle (the experiment was carried out 3 times, one typical result is represented).

An accumulation of ROS was previously observed in *A. thaliana* plants displaying increased cuticular permeability such as mutants defective in cuticle biosynthesis or in the formation of abscisic acid (ABA) [10]. Therefore we have determined if the SMS-stimulated leaves underwent a change in cuticular permeability. The permeability of the cuticle of SMS-stimulated leaves was assessed using various diagnostic tests. SMS-treated leaves displayed an increase in cuticular permeability as collectively indicated by increased chlorophyll leakage, retention of toluidine blue as well as Calcofluor white staining (Figure 6A to C).

### SMS is not accompanied by changes in ABA levels

**Figure 7:**
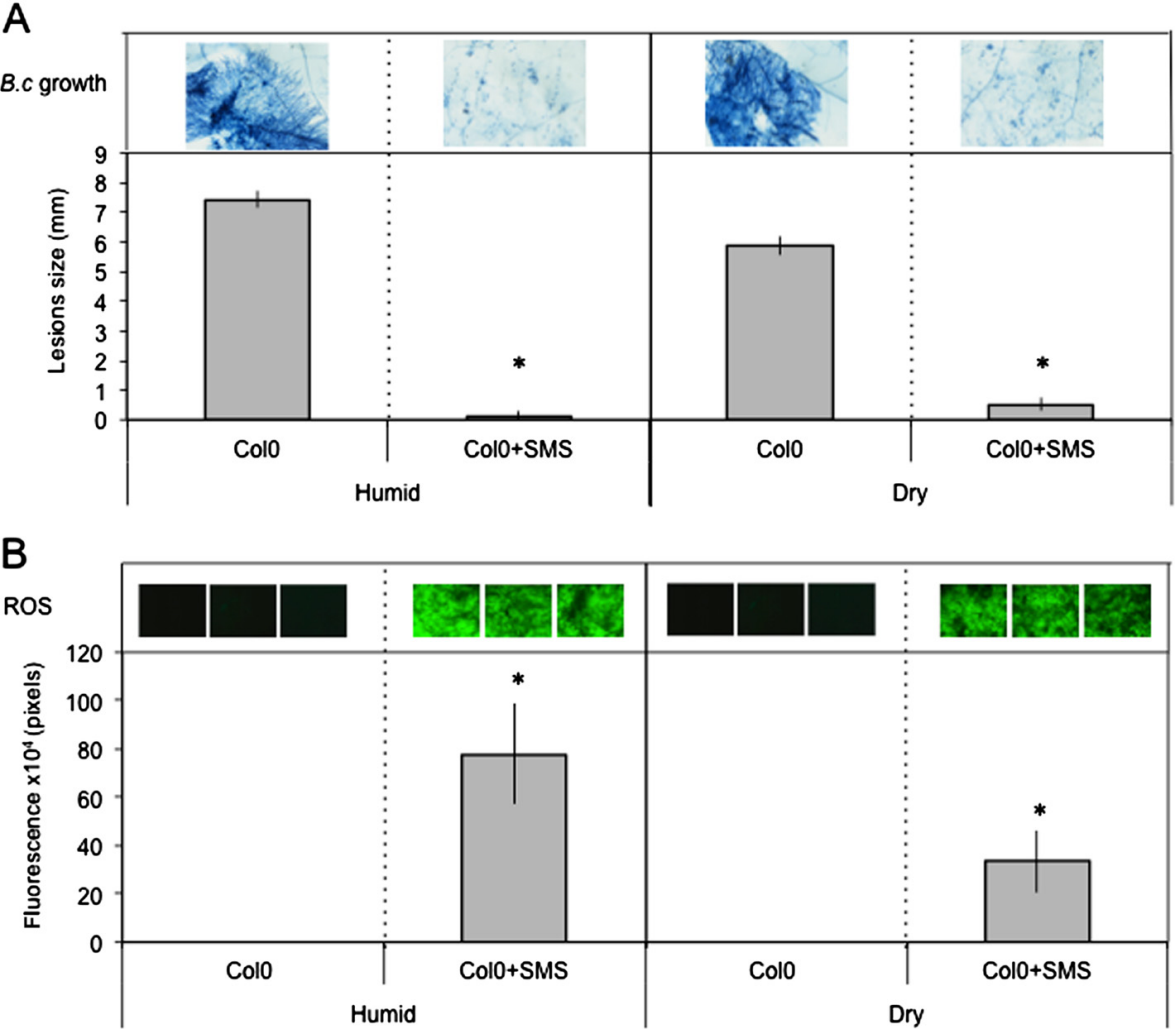
**The effect of humidity on resistance of *A. thaliana *Col0 to *B. cinerea *and ROS accumulation after SMS.** Leaves were stimulated by SMS and maintained for 1.5 h under high humidity in tightly covered well-watered trays (humid) or left in uncovered trays (dry) at room conditions prior to inoculation with *B. cinerea* and ROS detection. **(A)** Resistance to *B. cinerea* (n = 64; ±SE). One representative picture of the growth of *B. cinerea* was placed above each histogram as a visual illustration (trypan blue staining was carried out 3 days after inoculation). **(B)** Quantification of ROS production; three representative images of the fluorescent leaf surface were placed above each histogram as a visual illustration (n = 16; ±SD). Asterisks indicate statistically significant differences between treated samples and non-treated samples in humid and dry conditions, *T*-Test (p < 0,01).

Wound-induced resistance to *B. cinerea* is lost when wounded plants are not maintained under a humid environment (in covered trays). This loss is caused by ABA, the level of which increases under dry conditions (trays uncovered) [10]. In accordance, mutants impaired in ABA were fully resistant after wounding, both when plants were maintained under humid or dry environments [10]. Interestingly, increase in resistance and production in ROS were observed whether plants were maintained at humid or dry conditions after SMS (Figure 7A and B). Consequently, we also determined possible changes in the level of ABA. No changes were observed between the levels of ABA after SMS in plants maintained under humid or dry conditions (Additional file 3). This marks a clear difference between SMS and wound-induced resistance.

### SMS and the surface wax layer

**Figure 8:**
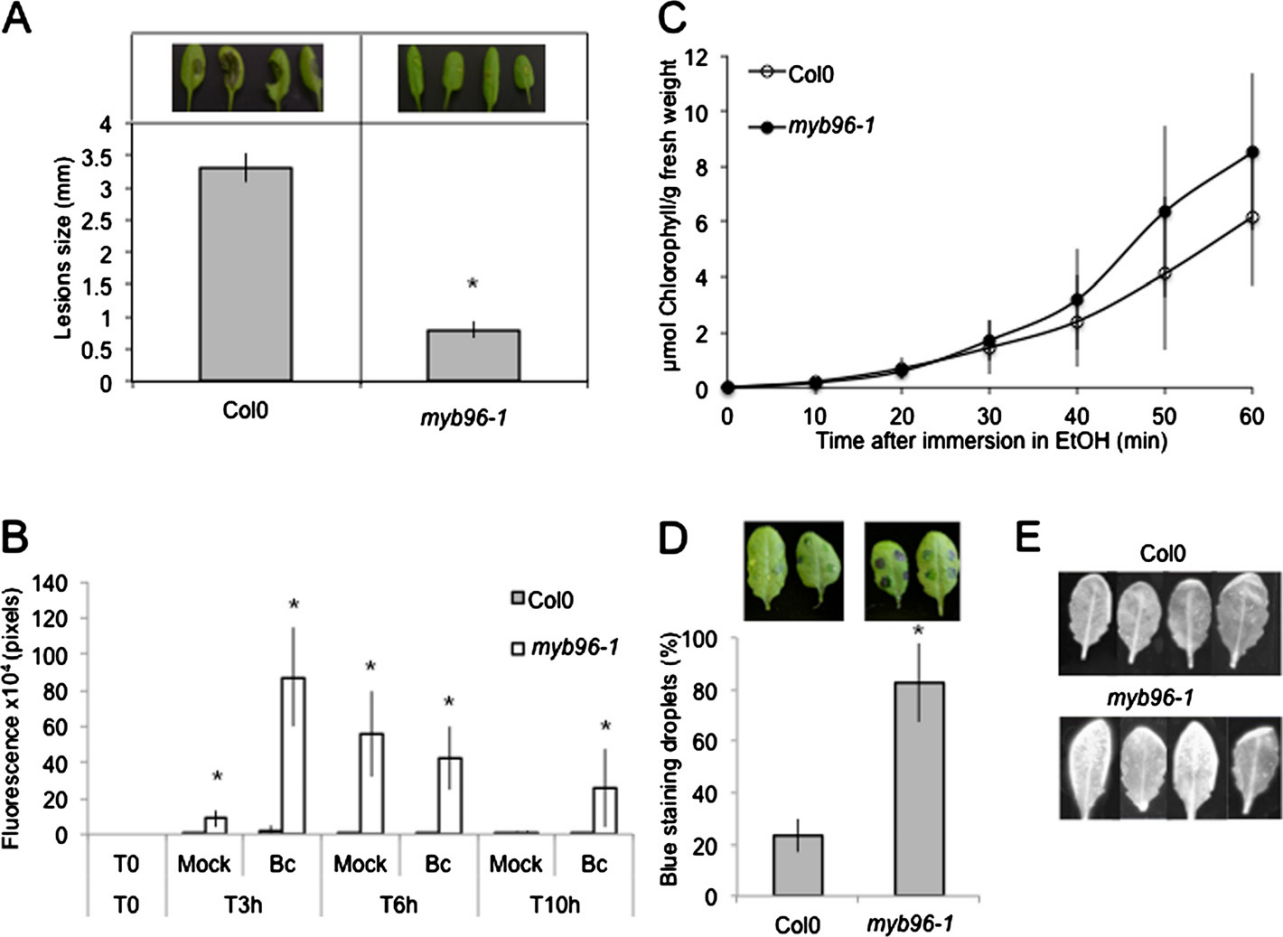
**Resistance to *B. cinerea *and ROS production in waxless *myb96KO *mutants. (A)** Resistance to *B. cinerea* in *myb96-1* mutant and wild type Col0 plants. Four representative pictures of necrosis due to *B. cinerea* are illustrated above each histogram. After *B. cinerea* inoculation, all plants were kept under humid conditions (n = 128; ±SE). The experiment was repeated twice with similar results. Asterisks indicate statistically significant differences between Col0 and *myb96-1* mutant, *T*-Test (p < 0,01). **(B)** Quantification of ROS production at 3, 6, 10 h after inoculation with *B. cinerea* (*Bc*) or mock treatment in *myb96-1* mutants compared to Col0. After treatment, all plants were kept under humid conditions (n = 6; ±SD). Asterisks indicate statistically significant differences between Col0 and *myb96-1* mutant after *B. cinerea* inoculation or mock treatment, *T*-Test (p < 0,01). **(C)** Permeability of the cuticle in WT Col0 leaves compared to *myb96-1* mutant. Leaves were placed in ethanol and the release of chlorophyll was followed over time (n = 3; ±SD). The experiment was carried out 3 times, one typical result is represented. **(D)** Permeability of the cuticle as determined by the toluidine blue test. The blue stain that remains attached to the cell wall is indicative of a permeable cuticle. The percentage of droplets stained in blue relative to the total inoculated droplets was calculated (n = 3; 90 droplets per experiment; ±SD). Two representative pictures of leaves stained with toluidine blue were placed above each histogram as a visual illustration. Asterisks indicate statistically significant differences between Col0 and *myb96-1* mutant, *T*-Test (p < 0,01). **(E)** Permeability of the cuticle as determined after Calcofluor white staining and viewing under UV light. Calcofluor retention by the cellulose is indicative of a permeabilized cuticle (the experiment was carried out 3 times, one typical result is represented).

Since SMS likely perturbs the waxy surface of the plant without much damage to the underlying cells (Figures 4 and 5A), we explored the importance of the wax layer on the leaf surface. We have used the *myb96-1* mutant affected in the transcription factor MYB96, involved in the biosynthesis enzymes condensing very-long-chain fatty acids involved in cuticular wax biosynthesis [19]. Untreated *myb96-1* mutants displayed increased resistance to *B. cinerea* (Figure 8A). The ROS response of *myb96-1* mutants to *B. cinerea* was much faster than in the wild type since green fluorescence was detected already 3 hours after inoculation with *B. cinerea* (Figure 8B). The *myb96-1* mutant also displayed a slight modification of permeability as indicated by the toluidine blue and Calcofluor white test, but this modification was not detected with the chlorophyll leakage test (Figure 8C-E). Thus, *myb96-1* altered in the wax layer displays a somewhat similar syndrome (increased resistance, ROS and a partial increase of permeability) although less obvious that the wild type leaves after SMS treatment.

### SMS and leaf diffusates

**Figure 9:**
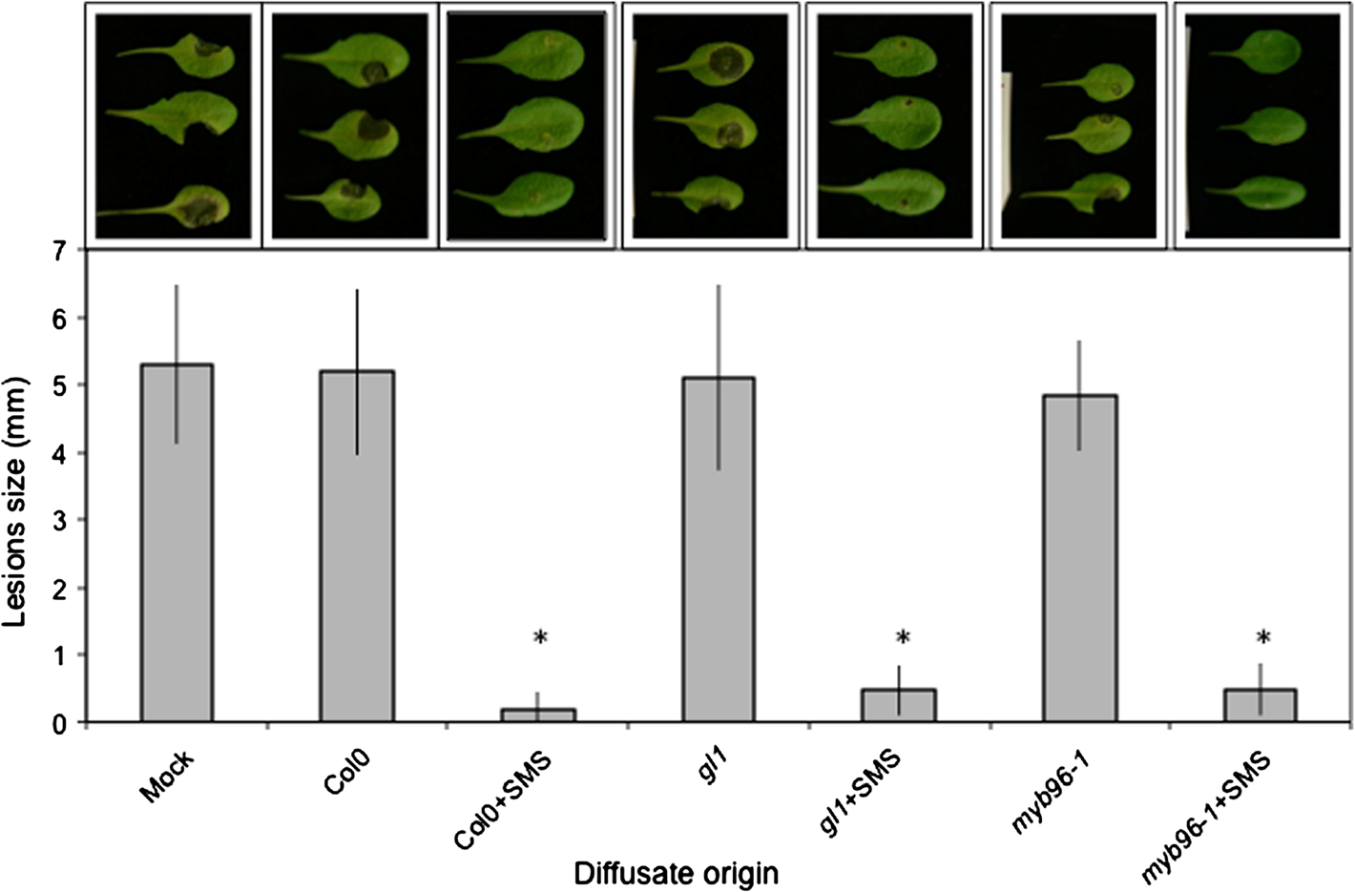
**Release of biologically active diffusates from leaves.** Resistance of WT Col0 plants inoculated with *B. cinerea* mixed with ¼ PDB (mock control) and with diffusates of non-treated and SMS treated Col0, *gl1* and *myb96-1* mutants. Tree representative pictures of necrosis caused by *B. cinerea* were placed above each histogram as a visual illustration. Asterisks indicate statistically significant differences between SMS treated and non-treated plants compared to mock control, *T*-Test (p < 0,01).

Changes in the permeability of the cuticle were shown to be associated with the leakage of diffusates that prevent the development of *B. cinerea in vitro* and *in vivo*[15,20]. We have tested if bioactive diffusates can be obtained from leaf surfaces of SMS-treated WT or *myb96-1* plants. SMS applied to *gl1* and *myb96-1* mutants was similarly effective as on SMS-treated WT plants (Figure 9). Without SMS, diffusates collected from the surfaces of both *gl1* and *myb96-1* plants were inactive similarly to those from WT plants. Thus SMS acted on leaf surfaces in a comparable way in plants or mutants displaying increased cuticular permeability [15,20].

## Discussion

Wounding inflicted by clamping leaves with forceps or puncturing with a needle induces a strong immunity of *A. thaliana* to *B. cinerea*[7]. In this study, we have explored the effect of softer forms of mechanical stimulation on the resistance of *A. thaliana* to *B. cinerea*. In particular, we have observed that a gentle mechanical stimulus applied to the surface of the leaf induced a transient and localized resistance to *B. cinerea*. Plants are known to be equipped with a sensitive and discriminative sensory system for the detection of molecular patterns generated by pathogens (pathogen- or microbe-associated molecular patterns, PAMPs or MAMPs; damage-associated molecular patterns, DAMPs) [21]. Here we show that a gentle mechanical stress can also be perceived in a differentiated way and lead to specific plant responses that include resistance against a virulent necrotrophic fungus.

How does SMS compare to wounding? Overall, the results on SMS-induced resistance to *B. cinerea* overlap with wound-induced resistance of *A. thaliana* leaves [10]. The results presented here further the published observations on wounding by showing that a soft mechanical friction of the surface layer without wounding the underlying cell is already enough to induce resistance. The absence of overt cellular breakage after SMS is supported by the absence of change in the levels of ABA after SMS under dry or humid conditions (Additional file 3) and the observation of leaf surfaces of WT and glabrous mutants after SMS (Figures 4 and 5A). Despite this, SMS-treated plants as well as the waxless mutant display an increased permeability to toluidine blue or Calcofluor white, indicating that the cuticular barrier is affected to a certain extent. The altered cuticular permeability might allow the diffusion of a bioactive molecule(s) observed in SMS-treated plants (Figure 9), similarly as in cuticle-defective mutants [15]. Both wounding and SMS lead to a rapid and important release of ROS. A number of reports have associated mechanical stress with an increased production of ROS [22-25]. For example, rubbing tomato plant internodes results in a rapid and lasting accumulation of H_2_O_2_[23]. ROS are well known for their effect as intracellular signals and were shown to be involved in the activation of defenses in response to biotic and abiotic stress [26]. SMS-induced resistance to *B. cinerea* still takes place in *atrbohD*, *atrbohF* and *atrbohD*/*F* mutants of NADPH oxidoreductase making it unlikely that these NADPH oxidoreductases are involved (Additional file 1). It cannot be excluded that some other Arabidopsis NADPH oxidoreductases are involved. This is similar to results obtained with these mutants after wounding [10]. In addition, SMS- like wound-induced resistance are both independent of JA signaling (Figure 3) [7].

How is SMS perceived by the plant? The results presented here lend themselves to a similar interpretation as the studies on resistance to *B. cinerea* observed in *A. thaliana* after wounding or in plants with defective cuticles [15,20]. SMS might modify the plant surface making it more permeable thus allowing a better transfer of DAMPs (possibly produced by the disturbance of the surface upon SMS) or MAMPs (from *B. cinerea*) through the cell wall into the cell where they are recognized by adequate receptors. The changes in cuticular permeability (Figure 6), the ROS production (Figure 1) and the diffusion of bioactive molecule(s) through the surface (Figure 9) are all hallmarks of such a scenario. The slightly increased permeability of *myb96-1* shown in 2 permeability tests (toluidine blue and Calcofluor white, Figure 8D-E) seems to be sufficient to allow for faster ROS production after inoculation and an increased resistance but not enough to allow sufficient leakage of active diffusates. Recent observations have shown a strong link between the presence of ROS and resistance to *B. cinerea* after wounding, and the data presented here agree with these conclusions. In fact, ROS accumulation and resistance after wounding were shown to depend on calcium changes and all occur at the same location further supporting this hypothesis (Beneloujaephajri et al., 2013, submitted). But the nature of the elicitors of these and corresponding receptors remain to be determined.

Sensing of stretch (or touch) by mechano-sensitive proteins, for example by stretch-activated channels in the membrane might be another way SMS is perceived and transduced. A possible model is that mechanical stimulus at the surface of the cell stretches such channels initiating a calcium flux [4,16,27,28]. The SMS-induced transient burst of calcium (Figure 2A and B) and the induction of genes (Figure 2C) that were previously associated with the perception of mechanical stimuli [29] would argue in favor of this scenario. But SMS like cutinase- or wound-induced resistance to *B. cinerea* are independent of JA signaling [7,20] (Figure 3), an observation that would differentiate SMS from a recent study on induced resistance to *B. cinerea* induced by leaf bending [30]. Leaf bending (ten times) also referred to as gentle touch is only accompanied by a ca 30% reduction in lesion after inoculation with *B. cinerea* and is JA-sensitive [30]. This contrasts with the present results where SMS was observed to lead to a full immunity to *B. cinerea* that is insensitive to JA. Experiments would now be needed with mutants blocked in mechano-sensitive touch receptors to differentiate between these pathways. It is most likely that SMS also leads to a major cellular reorganization such as that described by Hardham and colleagues (2008) [31]; the cellular details of the perception and attending mechanisms await now further studies.

## Conclusion

**Figure 10:**
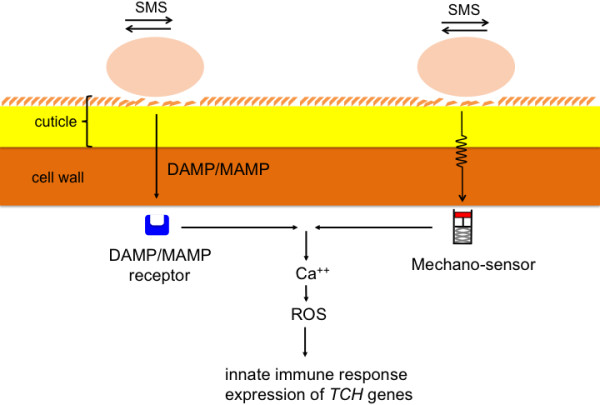
**Models of SMS-induced resistance in *A. thaliana.*** The plant might either recognize DAMPs or MAMPs following SMS. Such molecules are recognized by specific receptors and lead to changes in calcium and ROS with subsequent induction of defenses. Alternatively, the plant might sense the effect of SMS by membrane-bound mechano-sensors that lead to calcium changes, ROS and resistance.

Wounding and SMS exemplify how plants can react to a situation where in principle they might become more vulnerable. They rely on the deployment of a stress response that includes rapid changes in calcium levels and the release of active molecules such as ROS that subsequently lead to the activation of defense reactions exemplified by a strong resistance against the virulent *B. cinerea*. Interestingly, SMS is not associated with wounding and a modification in the wax layer is enough to produce this syndrome. The fact that SMS is also leading to the induction of so-called touch genes leaves two possible scenarios open: i) modifications of the plant surface by SMS lead to a facilitated perception of DAMPs or MAMPs by membrane receptors with subsequent activation of defenses or ii) SMS is perceived by mechano-sensors that subsequently initiate resistance (Figure 10). Overall, these results highlight the remarkable ability of plants to sense external mechanical stimuli and activate a powerful defense response.

## Methods

### Plant maintenance

*Arabidopsis thaliana* was grown on a pasteurized soil mix of humus and Perlite (3:1) in a growth chamber with a 12 h day/night photoperiod at 21°C/19°C, with a light intensity of 100 μE m^−2^ sec^−1^ and with a relative humidity of 60-70%. WT plants were the Arabidopsis accession Col0 obtained from the Arabidopsis Biological Research Center (Colombus, OH, USA). The Arabidopsis mutant referred to as *gl1* was in the Col0 background [18]. The *Arabidopsis* mutant *myb96-1* was in the Col0 background and was previously described [19]. The *Arabidopsis* mutants *dde 2.2*, *opr3*, *coi 1.16* were in the Col0 background.

#### Culture of *B. cinerea*, inoculation, staining of hyphae and SMS treatment

*B. cinerea* strain BMM, provided by Brigitte Mauch-Mani (University of Neuchâtel, Switzerland), were grown on Difco Potato Dextrose Agar (PDA) 39 g l^−1^ (Becton Dickinson, http://www.bd.com). Spores were harvested in water and filtered through glass wool to remove hyphae. Before inoculation, spores were diluted in ¼ strength Difco Potato Dextrose Broth (PDB) at 6 g l^−1^ (Becton Dickinson, http://www.bd.com) to the final concentration of 5 × 10^4^ spores ml^-1^. Six μl of spore suspension were deposited on leaves of 4-week-old plants. Lesion diameter was measured 3 days after inoculation using the digital caliper series 500 (Mitutoyo, http://www.mitutoyo.com). Data were integrated via the software for metrology IBREXDLL (IBRit, http://www.ibr.com). The inoculated plants were kept 3 days under high humidity (covered trays) in the growth chamber. Fungal structures and dead plant cells were stained by boiling inoculated leaves for 5 min in a solution of alcoholic lactophenol trypan blue. Stained leaves were extensively cleared in chloral hydrate (2.5 g ml^−1^) at room temperature by gentle shaking, and then observed using a Leica DMR microscope with bright-field settings.

Plant leaves were gently rubbed between thumb and forefinger without pressing with the thumb. For the time course experiment the SMS treatment was repeated 1, 5, 7 and 10 successive times, for the others experiments the SMS treatment was repeated 10 successive times. For SMS treatment of entire leaves, the SMS treatment was carried out on both sides of the main vein. SMS treatment leaves were incubated in covered trays at high humidity (referred to as humid conditions); in some cases the trays were left uncovered after SMS treatment (referred to as dry conditions) at the same laboratory conditions. Inoculation with *B. cinerea* was performed within 10 min after SMS treatment, by placing a droplet of spores on the SMS-treated site.

For the collection of diffusates, 8 μl of ¼ PDB were incubated for 24 h on non-treated and SMS-treated WT and mutants leaves. Leaf diffusates were collected and mixed with *Botrytis cinerea* spores to the final concentration of 5 × 10^4^ spores ml^-1^. The WT plants were inoculated in the same conditions as previously described.

#### Detection of ROS

ROS were detected using the fluorescent probe 5-(and-6)-carboxy-2’,7’-dichloro dihydrofluorescein diacetate (DCF-DA) (Sigma-Aldrich, http://www.sigmaaldrich.com) as previously described [10] . SMS treated- and non-treated leaves were vacuum-infiltrated (3 × 3 min) in 60 μM of DCF-DA in a standard buffer (1 mM KCl, 1 mM MgCl_2_, 1 mM CaCl_2_, 5 mM 2-morpholinoethanesulfonic acid adjusted to pH6.1 with NaOH) [32]. Leaves were then rapidly rinsed in DCF-DA buffer and observed using a Leica DMR epifluorescence microscope with a GFP filter set (excitation 480/40 nm, emission 527/30 nm) (Leica, http://www.leica.com). Microscope images were saved as TIFF files and processed for fluorescence quantification with Image J version 1.45 (NIH). Software settings were kept the same for every image analyzed and the area of green fluorescence corresponding to ROS production was expressed in pixels.

#### Tests of cuticle permeability

Chlorophyll extraction and quantification was performed according to a previously described protocol [33]. Leaves were cut at the petiole, weighed and immersed in 30 ml of 80% ethanol. Chlorophyll was extracted in the dark at room temperature with gentle agitation. Aliquots were removed at 10, 20, 30, 40, 50 and 60 min after immersion. The chlorophyll content was determined by measuring absorbance at 647 and 664 nm and the micromolar concentration of total chlorophyll per gram of fresh weight of tissue was calculated from the following equation: (19.53 × (A647 nm) + 7.93 × (A664 nm))/g fresh weight. The toluidine blue test was carried out by placing 6 μl droplets of a 0.025% toluidine blue solution in ¼ PDB on the leaf surface. After incubation for 2 h, leaves were washed gently with distilled water to remove excess of the toluidine blue solution from leaves. For staining with Calcofluor white, leaves were bleached in absolute ethanol overnight, equilibrated in 0.2 M NaPO_4_ (pH 9) for 1 h, and incubated for 1 min in 0.5% Calcofluor white in 0.2 M NaPO_4_ (pH 9). Leaves were rinsed in NaPO_4_ buffer to remove excess of Calcofluor white and viewed under UV light on a GelDoc 2000 system (Biorad, http://www.biorad.com).

#### RNA extraction and real time RT-PCR

The non-treated and SMS-treated leaves (10X) were ground. RNA was prepared using the Trizol reagent containing 38% saturated phenol, 0.8 M guanidine thiocyanate, 0.4 M ammonium thiocyanate, 0.1 M sodium acetate and 5% glycerol. RNA (1 μg) was then retrotranscribed into cDNA (Omniscript® RT kit, Qiagen, http://www.qiagen.com). RT-PCR was performed using Sensimix™ SYBR Green Kit (Bioline, http://www.bioline.com). Gene expression values were normalized to expression of the plant gene At4g26410, previously described as a stable reference gene [17]. The primers used were TCH3fw 5′- TCAAGGTCAGGGTCAAGTGC; TCH3rev 5′- TTGGCGAAGCGATGATATTGC; TCH4fw 5′- GAAACTCCGCAGGAACAGTC; TCH4rev 5′- TGTCTCCTTTGCCTTGTGTG; CML24fw 5′- GAGTAATGGTGGTGGTGCTTGA; CML24rev 5′- ACGAATCATCACCGTCGACTAA; CML39fw 5′- GATTGCATTACTCCGGGGAG; CML39rev 5′- GAGGGCGAACTCATCAAAGC.

#### Detection of calcium

The monitoring of the cytoplasmic calcium concentrations change was performed using transgenic *A. thaliana* plants expressing aequorin under the control of the cauliflower mosaic virus promoter 35S (gift from Marc Knight, Durham University). Leaves from 4 weeks old aequorin expressing plants were incubated overnight in 10 μM of coelenterazine (CTZ) in the dark to allow the binding between CTZ and aequorin. Basal level and stability of the luminescence signal before SMS treatment were then assessed by introducing the leaves in the luminometer (Sirius single tube luminometer, Berthold detection system, http://www.berthold-ds.com) where luminescence values were immediately scored every 3 seconds for one minute. After this time, 5 events of SMS were applied to the leaves directly in the luminometer and reading was carried out for three minutes. The luminescence was detected by using FB12 Sirius PC software.

#### Time-lapse Ca^2+^ imaging

Whole leaves of 16- to 21-day-old plants expressing cytoplasmic localized cameleon YC3.6 were placed in an open top chamber [34]. Leaves were imaged *in vivo* by an inverted fluorescence microscope Nikon Ti-E (Nikon, JP, http://www.nikon.com) with CFI planfluor 4× A.N.0,13 dry objective. Excitation light was produced by a fluorescent lamp Prior Lumen 200 PRO (Prior Scientific, UK) at 440 nm (436/20 nm) for Cameleon. Images were collected with a Hamamatsu Dual CCD Camera ORCA-D2 (Hamamatsu, Photonics, JP). The FRET CFP/YFP optical block A11400-03 (Emission 1 483/32 nm for CFP and Emission 2 542/27 nm for cpVenus with a dichroic mirror 510 nm) (Hamamatsu, Photonics, JP) was used for the simultaneous CFP and cpVenus acquisitions. Exposure time was 400 ms with a 2 × 2 CCD binning and images where acquired every 2 sec. Filters and dichroic mirror were purchased from Chroma (Chroma Technology Corporation, USA). The NIS-Element (Nikon, JP) was used as platform to control microscope, illuminator, camera and post-acquisition analyses. The fluorescence intensity was determined over regions of interest (ROIs) that correspond to the SMS-treated site. The SMS treatment was made 1 time. Due to the size of the imaged areas, the background was not subtracted. For cameleon analysis cpVenus and CFP emissions of the analyzed ROIs were used for the ratio (R) calculation (cpVenus/CFP) and normalized to the initial ratio (R0) and plotted versus time (ΔR/R0).

#### Scanning electron microscopy

SMS-treated and non-treated surfaces leaves were viewed with an S-3500 N variable pressure scanning electron microscope from Hitachi (http://www.hitachi.com), equipped with a cold stage.

## Competing interest

The authors declare that they have no competing interests.

## Authors’ contributions

LB, FL: conceptualization of the experiments, ROS staining and quantification, *B. cinerea* infections on wild type and various mutants, trypan blue staining, permeability tests, critical revision of the manuscript. FL, JPM: SEM observations. EAM: Analysis of ABA. MS: experiments on leaf diffusates, critical revision of the manuscript. MB, AC: time-lapse calcium imaging, critical revision of the manuscript. SL: quantification of touch gene expression, critical revision of the manuscript. JPM: conceptualization of the experiments, manuscript writing, critical revision of the manuscript. All authors read and approved the final manuscript.

## Supplementary Material

Additional file 1 **Resistance to *B. cinerea *in *NADPH oxidase *mutants after SMS.** Leaves of *NADPH oxidase* mutants treated with SMS (10×) prior to inoculation with *B. cinerea*. SMS-induced resistance to *B. cinerea* were still detected in treated leaves in *atrboh D* and *atrboh F* as well as in the double mutant *atrboh D/F* (n = 64; ±SE)*.* After SMS, all plants were kept under humid conditions. Asterisks indicate statistically significant differences between non-treated and SMS-treated plants for Col0 and each mutant, *T*-Test (p < 0,01). Click here for file

Additional file 2 **Resistance to *B. cinerea *in response to SMS is transient.** Leaves were treated with SMS and inoculated with *B. cinerea* at the times indicated (in h). Lesion diameters were measured 3 days after infection (n = 64; ±SE). Asterisks indicate a significant difference from the non-treated (NT) (p < 0.05). Click here for file

Additional file 3 **ABA accumulates after SMS under dry conditions.** SMS-treated leaves were maintained for 1.5 h under high humidity in tightly covered well-watered trays (humid) or in uncovered trays at room conditions (dry) prior to measurement of ABA. Following the method of Schmelz et al. (2004) [35], ABA was measured in ng mg^−1^ fresh weight of plant tissue in non-treated or treated leaves, incubated under humid or dry conditions (n = 5; ±SD). Asterisks indicate statistically significant differences between treated samples and non-treated samples in humid and dry conditions, *T*-Test (p < 0,01). Click here for file
