# Supplementary figures and images for: Perception of soft mechanical stress in Arabidopsis leaves activates disease resistance
Source: BMC Plant Biol. 2013 Sep 13;13:133. doi: 10.1186/1471-2229-13-133 (PMC3848705; doi:10.1186/1471-2229-13-133)

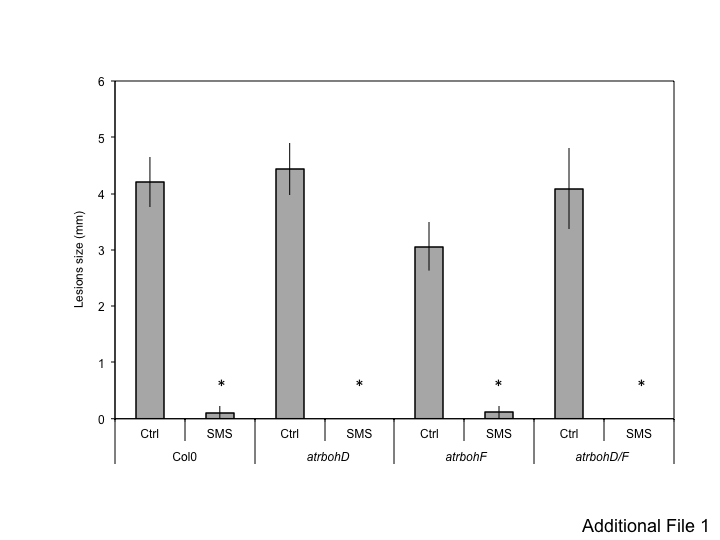

Supplement: Additional file 1 — Resistance to B. cinerea in NADPH oxidase mutants after SMS. Leaves of NADPH oxidase mutants treated with SMS (10×) prior to inoculation with B. cinerea. SMS-induced resistance to B. cinerea were still detected in treated leaves in atrboh D and atrboh F as well as in the double mutant atrboh D/F (n = 64; ±SE). After SMS, all plants were kept under humid conditions. Asterisks indicate statistically significant differences between non-treated and SMS-treated plants for Col0 and each mutant, T-Test (p < 0,01). [file 1471-2229-13-133-S1.tiff]

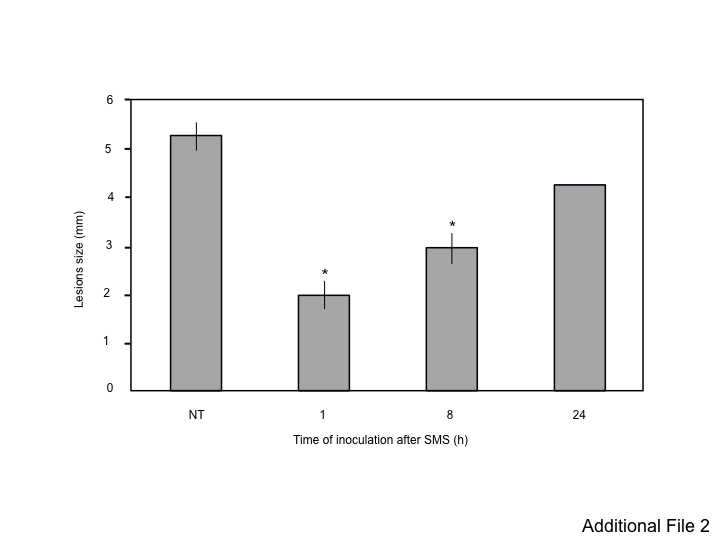

Supplement: Additional file 2 — Resistance to B. cinerea in response to SMS is transient. Leaves were treated with SMS and inoculated with B. cinerea at the times indicated (in h). Lesion diameters were measured 3 days after infection (n = 64; ±SE). Asterisks indicate a significant difference from the non-treated (NT) (p < 0.05). [file 1471-2229-13-133-S2.tiff]

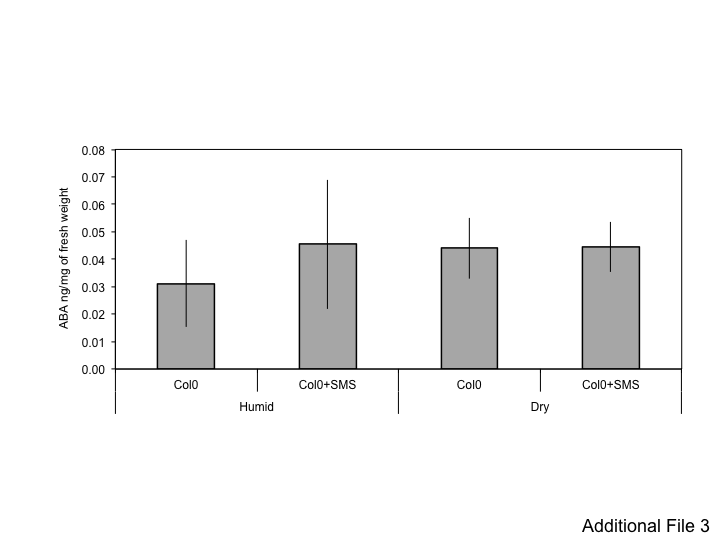

Supplement: Additional file 3 — ABA accumulates after SMS under dry conditions. SMS-treated leaves were maintained for 1.5 h under high humidity in tightly covered well-watered trays (humid) or in uncovered trays at room conditions (dry) prior to measurement of ABA. Following the method of Schmelz et al. (2004) [35], ABA was measured in ng mg−1 fresh weight of plant tissue in non-treated or treated leaves, incubated under humid or dry conditions (n = 5; ±SD). Asterisks indicate statistically significant differences between treated samples and non-treated samples in humid and dry conditions, T-Test (p < 0,01). [file 1471-2229-13-133-S3.tiff]
